# Novel thalidomide analogues display anti-angiogenic activity independently of immunomodulatory effects

**DOI:** 10.1038/sj.bjc.6600607

**Published:** 2002-11-04

**Authors:** K Dredge, J B Marriott, C D Macdonald, H-W Man, R Chen, G W Muller, D Stirling, A G Dalgleish

**Affiliations:** Division of Oncology, St. George's Hospital Medical School, Tooting, London SW17 0RE, UK; Celgene Corporation, Warren, New Jersey, USA

**Keywords:** thalidomide analogues, anti-angiogenic, immunomodulatory, IMiDs, SelCIDs

## Abstract

The anti-tumour effects of thalidomide have been associated with its anti-angiogenic properties. Second generation thalidomide analogues are distinct compounds with enhanced therapeutic potential. Although these compounds are beginning to enter trials for the treatment of cancer there is very little information regarding the anti-angiogenic activity of these clinically relevant compounds. Furthermore, it is not known how the various immunomodulatory activities of these compounds relate to anti-angiogenic activity. In this study we assessed the anti-angiogenic activity of compounds from both IMiD™ and SelCID™ classes of analogues using a novel *in vitro* multicellular human assay system and the established rat aorta assay. Our results show that both the IMiDs and SelCIDs tested are significantly more potent than thalidomide. The anti-angiogenic potency of the analogues was not related to inhibition of endothelial cell proliferation, nor their TNF-α/PDE type 4 inhibitory properties. However, anti-migratory effects *in vitro* and inhibition of tumour growth *in vivo* was observed with the analogue IMiD-1 (clinically known as REVIMID™). Our results show that anti-angiogenic activity spans both currently defined classes of thalidomide analogue and is not related to their previously described immunomodulatory properties. Identification of the differential effects of these compounds will enable targeting of such compounds into the appropriate clinical setting.

*British Journal of Cancer* (2002) **87**, 1166–1172. doi:10.1038/sj.bjc.6600607 www.bjcancer.com

© 2002 Cancer Research UK

## 

Angiogenesis is the formation of new blood vessels by pre-existing endothelial cells (EC) and plays an important role in tumour growth and progression (Folkman, 1971). The ability to attract new vasculature from the host is thought to be a characteristic feature of tumour cells. During vessel co-option tumours will initially exploit the host vasculature for survival and this coincides with host vasculature regression (Griffioen and Molema, 2000). Ongoing tumour cell growth will subsequently lead to the initiation of angiogenesis (Holash *et al*, 1999). Without blood vessels tumours cannot grow beyond a critical size or metastasise to another organ and therefore inhibition of such processes could be a strategy for tumour arrest (Carmeliet and Jain, 2000). Angiogenesis depends mainly on proper activation, proliferation, adhesion, migration and maturation of EC (Griffioen and Molema, 2000). Therefore, inhibition of EC growth, adhesion and migration, matrix metalloproteinase and growth factor expression are putative anti-angiogenic targets.

Thalidomide has previously been shown to inhibit angiogenesis in experimental models (D'Amato *et al*, 1994; Kenyon *et al*, 1997). It has also been found inhibit microvessel formation when co-incubated with rabbit or human microsomes, but not rat microsomes or thalidomide alone (Bauer *et al*, 1998). The anti-angiogenic activity of thalidomide is likely to be a contributing factor for its anti-tumour effects in MM (Singhal *et al*, 1999) for which it has been granted FDA approval. Furthermore, anti-angiogenic activity has provided the rationale for its use in the treatment of other cancers.

The side effects associated with clinically effective thalidomide treatment has led to the design and synthesis of more potent analogues with reduced toxicity. These are being characterised and currently segregate into at least two distinct classes; Selective Cytokine Inhibitory Drugs (SelCIDs), which are PDE4 inhibitors, and Immunomodulatory Drugs (IMiDs) whose mechanism(s) of action remain unknown. Both groups contain compounds with potent anti-TNF-α activity although T cell costimulatory activity is limited to the IMiD class (Corral *et al*, 1999; Marriott *et al*, 2001). However, very little is known concerning their anti-angiogenic properties. One SelCID analogue (CC-1069) has previously been shown to inhibit human EC proliferation *in vitro* to a greater extent than thalidomide, suggesting that it may be a more potent anti-angiogenic agent (Moreira *et al*, 1999). In our study we set out to characterise the *in vitro* anti-angiogenic activity of both classes of analogue to determine whether this correlates with their immunomodulatory properties.

## MATERIALS AND METHODS

### Thalidomide and analogues

For the human angiogenesis assay thalidomide and various analogues were dissolved in DMSO (Sigma, Kent, UK) to obtain a stock solution of 1 mg ml^−1^. Further dilutions were made in warm culture medium immediately before use and 1 and 10 μg ml^−1^ of thalidomide or analogues were administered every 48 h. Final DMSO concentration was 0.05%. For the rat aorta assay, thalidomide analogues (Celgene, Warren, NJ, USA) were prepared in Tween 80 (Sigma, Kent, UK). Dilutions were made in culture medium immediately before use and 1 or 10 μg ml^−1^ of the analogues were administered every 48 h. The final Tween 80 concentration was 0.025%. For *in vivo* treatment experiments, mice were treated daily with 10 or 50 mg kg^−1^ IMiD-1 with 0.5% DMSO as vehicle (see Tumour challenge model).

### Human angiogenesis kit

A commercially available human angiogenesis kit, used for the assessment of angiogenesis *in vitro* (Bishop *et al*, 2000; Donovan *et al*, 2001; Terai *et al*, 2001; Drinkwater *et al*, 2002; Osugi *et al*, 2002) was provided by TCS Cellworks. The assay is supplied as growing cultures of HUVECs at the earliest stages of tubule formation in a 24 well plate format. The assay was carried out according to manufactures instructions with minor modifications. In brief, 0.5 ml^−1^ of fresh medium or medium plus drug preparations were added to the appropriate wells. A known angiogenic stimulator (VEGF at 5 ng ml^−1^) and inhibitor (suramin at 10 μg ml^−1^) were included as positive and negative controls respectively in addition to medium and DMSO controls and plates were incubated at 37°C. Drug administration was carried out every 48 h. On day 11, wells were fixed using ice cold 70% EtOH prior to incubation (RT) for 30 min. Blocking buffer was then added for 10 min. Endothelial cells were visualised by first staining with a primary sheep anti-human for von Willebrand factor antibody followed by a secondary donkey anti-sheep IgG horseradish peroxidase conjugate prior to colour development using a DAB metal substrate. The plate was washed with ddH_2_O (×3) and allowed to air dry overnight. Cultures were scored manually using a 25-point Chalkley eyepiece graticule (Graticules Ltd., Tonbridge, Kent, UK), previously described as a satisfactory method of assessing tumour vascularity (Fox *et al*, 1995). The extent of angiogenesis was assessed by counting the number of points on the chalkley graticule that landed on a tubule. The count was repeated 12 times for each well. The term ‘graticule unit’ was assigned to this measurement of tubule density.

### Rat aorta assay

Preparation of agarose culture wells and aortic explants were prepared as described originally Nicosia and Ottinetti, 1990, and subsequently modified by Stevenson *et al*, 1998. In brief, aortic explants were stored in DMEM/HAM F12. Agarose rings were obtained by punching two concentric circles in the sterile agarose gel (1.5%, w v^−1^) with specifically designed aluminium punches. Collagen type I from rat tail (Sigma) was dissolved overnight in glacial acetic acid and diluted to 4.3 mg ml^−1^ with 10% (v v^−1^) DMEM/HAM F12. The final collagen solution was obtained by mixing seven volumes of 4.3 mg ml^−1^ collagen with two volumes NaHCO_3_ (11.7 mg ml^−1^ in DMEM/HAM F12) and one volume of 10×MEM. Finally, the pH was adjusted to 7.4 with 0.1 M NaOH using phenol red as a visual indicator. Prior to embedding of explants in collagen gel, the bottom of each agarose well was coated with 200 μl collagen solution and allowed to set at 37°C. The wells were then filled (further addition of 400 μl) and explants were promptly orientated into the centre of the collagen gel. The plates were incubated overnight with serum-free medium before group allocation and drug treatment commenced. Drugs were prepared as described and diluted in DMEM/HAM F12 medium to be administered at each media change (48 h). Microvessel outgrowths in all cultures were quantified at each media change.

### Proliferation studies

Human umbilical vein endothelial cells (HUVEC) were purchased from TCS Cellworks Ltd. (Park Leys, Botolph Claydon, Bucks, UK) and cultured in large vessel EC basal medium and cell growth supplement (TCS Cellworks Ltd.). The cells were passaged between 3–5 times prior to use. The human endothelial-like EA.hy926 cell line, derived from the fusion of HUVEC with the A549 carcinoma cell line (Edgell *et al*, 1983), was kindly provided by Cora-Jean Edgell (NC, USA). The cells were cultured in DMEM with high glucose, 10% FCS and HAT. Both cell lines were harvested from subconfluent cultures and diluted to 4×10^4^ cells ml^−1^. Cells were seeded in 60 wells of a 96-well plate in a volume of 100 μl^−1^. One plate was used per test article. Plates were incubated for 1 h at 37°C and 5% CO_2_. Growth factors were added at a volume of 50 μl^−1^ with final concentrations of 10 ng ml^−1^. Thalidomide and its analogues were added at a volume of 50 μl^−1^ in final concentrations of 5, 10, 25, 50 μg ml^−1^. DMSO as used as a vehicle at a final concentration of 0.05%. Plates were incubated at 37°C with 5% CO_2_ for 96 h. During the last 18 h of culture 1 μCi^−1^ of ^3^H-thymidine was added per well. Plates were then stored at −20°C until analysis using a Microbeta counter (Wallac, UK).

### Wound assay for EA.hy926 cell migration

We tested IMiD-1 in the wound healing assay as described by Igura *et al*, 2001. Briefly, confluent monolayers of EA.hy.926 cells in 35-mm dishes were wounded with a rubber policeman and washed with PBS. Then the dishes were incubated in complete DMEM containing IMiD-1 or suramin for 16 h at 37°C in a 5% CO_2_ incubator. DMSO (0.05%) was used as control. The cell monolayers were fixed with absolute methanol and stained with Giemsa. The number of cells that had migrated from the edge of the wound in each 125×500 μm area of eight randomly chosen fields was counted. The results are presented as the average number of cells per field.

### Phosphodiesterase (PDE) type 4 assay

PDE type 4 purification from U937 cells was carried out using the method of Hill and Mitchell (1994). PDE activity was assayed, using a method described by di Santo and Heaslip (1993), in the presence of varying concentrations of thalidomide analogues. Each data point was carried out in duplicate with activity expressed as percentage of control. IC_50_ was determined from dose response curves derived from three independent experiments.

### Tumour challenge model

MF1 nude mice (nu nu^−1^) were purchased from Harlan UK. Mice were age and sex-matched for individual experiments. All animal experiments have been carried out with ethical committee approval. The ethical guidelines that were followed meet the standards set by the UKCCCR guidelines (Workman *et al*, 1998). CMT93 is an epithelial-like murine rectum polypoid carcinoma cell line with sarcomatous features derived from a C57/BL mouse (Franks and Hemmings, 1978). Mice were challenged subcutaneously with 200 μl of the murine colorectal cell line CMT93 (2×10^6^ per mouse). Once the tumours reached a size of 20 mm^3^, the potent anti-angiogenic analogue IMiD-1 was administered intraperitoneally daily at a concentration of 10 or 50 mg kg^−1^. Tumours were measured every 3–4 days, and volumes (in mm^3^) were calculated by the use of the formula (width [mm^2^]×length [mm]×0.52). The animals were observed daily, and those bearing large tumours were monitored carefully for any signs of discomfort. All mice were healthy and maintained weight as normal throughout the study. Histological samples were obtained using formalin-fixed, paraffin-embedded sections stained for haematoxylin and eosin and viewed under a microscope at a magnification of ×400.

### Statistics

One-way analysis of variance (ANOVA) followed by a *post hoc* Dunnett's *t*-test was used to analyse the data. A *P* value <0.05 was considered to be significant. Angiogenesis data are expressed as percentage of controls to allow intra-assay comparisons.

## RESULTS

### *In vitro* angiogenesis assays

**Figure 1:**
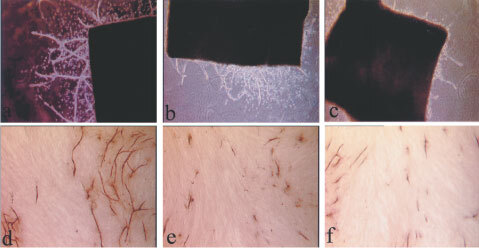
Illustrates the effect of a thalidomide analogue, IMiD-1 (**C**,**F**) and suramin (**B**,**E**) in comparison to their relative controls (**A**,**D**) on microvessel outgrowths in the rat aorta assay (top row) and on tubule development in the human angiogenesis model (bottom row).

In the human angiogenesis model, significant inhibitory effects (*P*<0.05) of IMiDs and SelCIDs were observed at 10 μg ml^−1^ (Figures 1

and 2

**Figure 2:**
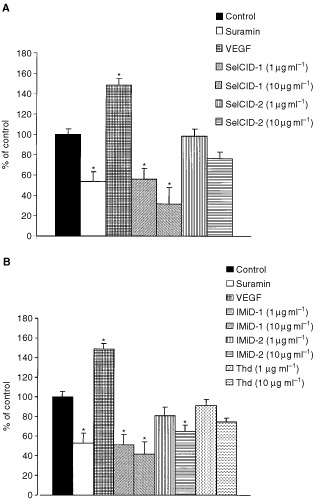
Inhibition of angiogenesis as demonstrated by the reduction of tubule development following co-culture for 11 days with SelCIDs (**A**) or with thalidomide or IMiDs (**B**) in the human angiogenesis model. The effect of suramin or VEGF on tubule development is also shown. . *=*P*<0.05 *vs* control (Dunnett's test).

). In particular, IMiD-1 and SelCID-1 were more potent than suramin at this concentration. Furthermore, these analogues showed a significant reduction in tubule development at 1 μg ml^−1^ (*P*<0.05). IMiD-2 and SelCID-2 were the least potent in terms of their anti-angiogenic activity with non-significant inhibition of tubule development at 10 μg ml^−1^. Moreover, only non-significant inhibition of tubule development was observed using thalidomide. As expected the pro-angiogenic effects of VEGF were readily apparent in this system.

**Figure 3:**
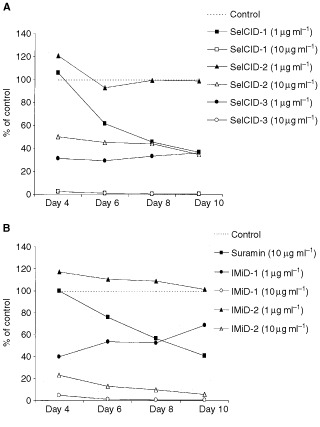
Inhibition of angiogenesis as demonstrated by the reduction in the number of microvessels observed following co-culture with SelCIDs (**A**) or with IMiDs (**B**) in the rat aorta angiogenesis model. The effect of suramin on microvessel development is also shown. *=*P*<0.05 *vs* control (Dunnett's test).

In the rat aorta assay, microvessels sprouted from the edges of explants in control groups by day 4 of culture. The number and length of the vessels increased with time until the experiments were terminated after 10 days of culture (Figures 1 and 3

). Suramin was found to significantly inhibit the number of microvessel outgrowths by day 6 at a concentration of 10 μg ml^−1^, previously determined to be its IC_50_ (data not shown). The SelCID derivatives also significantly inhibited angiogenesis in this model (Figure 3A). SelCID-1 displayed a similar pattern to suramin at 1 μg ml^−1^ while causing almost 100% inhibition at 10 μg ml^−1^ (*P*<0.05). However, whilst SelCID-3 showed potent inhibition at both concentrations, SelCID-2 had no effect at 1 μg ml^−1^ and only 50% inhibition at 10 μg ml^−1^.

The IMiD analogues also demonstrated activity as IMiD-1 (at 1 μg ml^−1^) significantly inhibited microvessel outgrowths by day 4 (*P*<0.05) and resulted in 100% inhibition at 10 μg ml^−1^ (Figure 3B). In contrast, IMiD-2 had no effect at 1 μg ml^−1^, although 90% inhibition was observed at the 10 μg ml^−1^ concentration by day 10. Thalidomide (at up to 50 μg ml^−1^) did not significantly inhibit microvessel development in the rat aorta assay (data not shown). In some experiments, drug withdrawal after 8 days followed by subsequent stimulation with VEGF (10 ng ml^−1^) for a further 6 days, resulted in microvessel outgrowths 98% of control in the 1 μg ml^−1^ groups and 76% of control in the 10 μg ml^−1^ groups (data not shown). This demonstrated that the analogues were non-toxic in this system.

### Endothelial cell proliferation and migration

**Figure 4:**
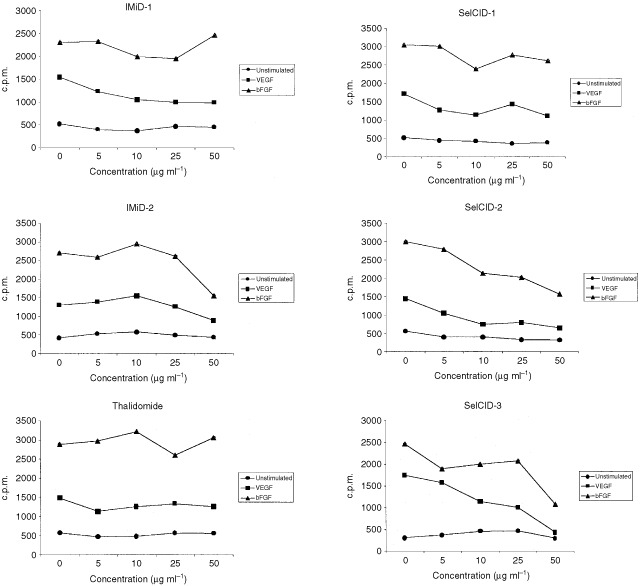
The effect of Thd, IMiDs and SelCIDs on bFGF and VEGF-induced HUVEC proliferative responses. The data is representative of three independent experiments.

In the proliferation studies, we found that SelCID-2 and 3 inhibited bFGF and VEGF-induced proliferation (*P*<0.05) of HUVECs and EA.hy926 (data not shown for EA.hy926) but SelCID-1 had no effect of proliferative responses (Figure 4

). Moreover, IMiD-2 inhibited VEGF (*P*<0.05) but not bFGF-induced proliferation at 50 μg ml^−1^, while thalidomide and the potent anti-angiogenic compound IMiD-1 did not significantly inhibit EC growth rates. Suramin did not inhibit proliferation at the concentrations tested. However, IMiD-1 and suramin did significantly inhibit the migration of EC (*P*<0.05) in the wound healing assay suggesting that both compounds may inhibit angiogenesis by interfering with normal migratory regulation.

### Tumour challenge model

**Figure 5:**
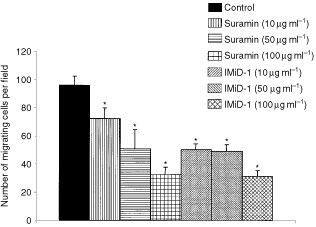
The effect of suramin and IMiD-1 on the migratory properties of EA.hy926 cells in an *in vitro* wound healing assay. Results are expressed as mean number of cells migrating per field. *=*P*<0.05 versus control (Dunnett's test).

In order to determine whether a thalidomide analogue with potent anti-angiogenic activity *in vitro* could be beneficial in a clinical cancer setting, we tested the most potent analogue IMiD-1, in an *in vivo* tumour challenge model. Daily administration of IMiD-1 (10 or 50 mg kg^−1^, i.p.) was found to significantly reduce the tumour growth rates (*P*<0.05) in nude mice and histological examination revealed drug-treated tumours to contain vast regions of necrotic tissue (Figure 5

).

### Immunomodulatory studies

The PDE4 and TNF-α inhibitory activity of thalidomide and its analogues are presented in Table 1

**Table 1:**
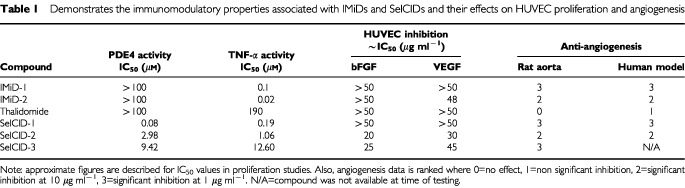
Demonstrates the immunomodulatory properties associated with IMiDs and SelCIDs and their effects on HUVEC proliferation and angiogenesis

. This data showed that PDE4 inhibition and TNF-α inhibition are not related to the ability to inhibit angiogenesis.

## DISCUSSION

This study is the first to characterise the anti-angiogenic properties of thalidomide analogues derived from the distinct IMiD and SelCID classes of compound. These groups of analogues have distinct immunological effects although they both inhibit TNF-α production by activated monocytes/macrophages. We have shown that compounds from both groups possess potent anti-angiogenic activity in a novel human system, confirmation of this was obtained using the established rat aorta assay which gave similar results. Anti-angiogenesis was observed by inhibition of microvessel/tubule development in a dose-dependent manner.

Results obtained using the human angiogenesis model showed that IMiD-1 and SelCID-1 were able to significantly inhibit angiogenesis even at the lowest concentration used (1 μg ml^−1^). IMiD-2 significantly inhibited tubule development only at the higher concentration (10 μg ml^−1^), whereas at this concentration SelCID-2 and thalidomide itself showed only non-significant inhibition. Verification of this assay system is supported by our data showing VEGF significantly increased angiogenesis in this assay while suramin inhibited angiogenesis by approximately 50%. Suramin is an established anti-angiogenic agent and has been used to treat patients with various cancers (Stein *et al*, 1989; Danesi *et al*, 1993; Small *et al*, 2000). Therefore, the anti-angiogenic potency of these compounds suggests that they may be of improved therapeutic value.

Thalidomide has minimal effects in the rat system, most probably due to species specificity. However, it has some effect in the human assay, although the analogues are clearly more potent in terms of their anti-angiogenic activity. Previously Bauer and colleagues (Bauer *et al*, 1998) have shown that thalidomide inhibited angiogenesis in the rat aortic angiogenesis assay only when used in combination with a microsomal preparation to allow for its metabolism. We have noted a similar effect in this system (data not shown). However, IMiD-1, SelCID-1 and SelCID-3 significantly inhibited angiogenesis in the rat aorta model at very low concentrations and in the absence of microsomes. In contrast, IMiD-2 and SelCID-2 inhibited microvessel development to a lesser extent (10 μg ml^−1^). Inhibition of angiogenesis in this system was a non-toxic effect, as drug withdrawal and subsequent stimulation of the aortic explants with VEGF led to the development of new microvessels (data not shown). In summary, the data generated using the novel human assay correlate with those obtained using the rat aorta assay. This is important in two respects; firstly, it enables *in vitro* testing of putative anti-angiogenic agents prior to their clinical assessment *in vivo* so that species specificity is not an issue. Secondly, there can be more confidence that anti-angiogenic activity previously characterised in the rat model is applicable to the human situation.

The analogues with potent anti-angiogenic activity (e.g. IMiD-1, SelCID-1) did not affect VEGF- or bFGF-induced HUVEC or EA.hy926 proliferation (Figure 4). However, CC-1069 (referred to here as SelCID-3) inhibited HUVEC proliferation at similar concentrations as previously described (Moreira *et al*, 1999). SelCID-2 and IMiD-2 also showed some anti-proliferative activity but concentrations required were higher to inhibit proliferation than anti-angiogenic activity. It is of interest to note that suramin did not inhibit proliferation of HUVECs at the concentrations tested, a finding consistent with previous data showing that 250 μg ml^−1^ of suramin is required to inhibit EC proliferation (Takano *et al*, 1994). This indicates that the anti-angiogenic effect observed does not correlate with antiproliferative effects on EC. However, suramin and IMiD-1 did significantly inhibit migration at concentrations relevant to their anti-angiogenic effects. Therefore, our data indicates that IMiD-1 may inhibit angiogenesis due to anti-migratory rather than anti-proliferative mechanisms.

**Figure 6:**
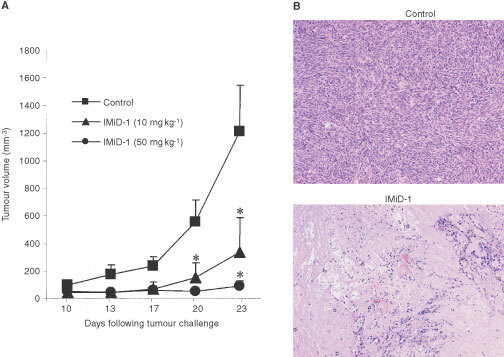
The effect of IMiD-1 on the growth rate of CMT93 colorectal tumour in nude mice (**A**). Data is representative of at least two independent experiments. *=*P*<0.05 *vs* control (Dunnett's test). Histological examination revealed that treatment with IMiD-1 (bottom picture) increased necrosis within the tumour (**B**). Magnification ×400.

In order to determine whether such activity would be applicable to a clinical cancer setting, we investigated the ability of the lead anti-angiogenic analogue IMiD-1, to inhibit tumour development *in vivo*. CMT93 tumour growth rates were significantly reduced in nude mice treated daily with IMiD-1 and these tumours were also found to have large central necrotic areas (Figure 6

). Since there is no direct drug effect on these cells *in vitro* (data not shown), we believe that the effect is mediated via inhibition of angiogenesis. This may be due to an anti-VEGF effect since IMiD-1 could inhibit the secretion of VEGF by CMT93 cells *in vitro* (data not shown). However, it was not possible to detect VEGF in serum from the mice.

An important observation of this study is that the anti-angiogenic properties of IMiDs and SelCIDs cannot be attributed to previously described functional effects. IMiDs or SelCIDs inhibit angiogenesis but do not inhibit EC proliferation. This is also apparent with other compounds such as thymidine phosphorylase whose anti-angiogenic activity is also independent of anti-proliferative activity (Liekens *et al*, 2001). The anti-angiogenic effect of IL-12 is exerted by triggering the secretion of IFN-γ, which presumably induces IP-10 and MIG (Duda *et al*, 2000) and not via the inhibition of endothelial cells (Voest *et al*, 1998). SelCIDs are also known to potently inhibit PDE4 enzymes resulting in the inhibition of TNF-α production (Muller *et al*, 1998; Marriott *et al*, 1998). However, we found no correlation between the IC_50_s for either PDE4 inhibitory or anti-TNF-α properties of these compounds and anti-angiogenesis. TNF-α is weakly angiogenic *in vivo* (D'Amato *et al*, 1994). Therefore, the anti-angiogenic activity of IMiDs cannot be attributed to their anti-TNF-α properties. Finally, IMiD-2 costimulates human T cells to a greater degree than IMiD-1 (Marriott *et al*, 2002) indicating mechanisms involved in T cell activation are not involved in determining anti-angiogenic activity.

The effects of SelCIDs and IMiDs in this study provide further evidence of the clinical potential of these novel compounds as anti-tumour drugs. IMiD analogues have been shown to induce myeloma cell growth arrest *in vitro* (Hideshima *et al*, 2000). We have also shown that a subgroup of SelCID analogues can induce apoptosis in myeloma cells and also a variety of solid tumour cell types *in vitro* and *in vivo* (Marriott *et al*, 2002). Since IMiD-1/REVIMID™ is currently under phase I/II clinical investigation in the treatment of end stage cancer patients (Marriott *et al*, 2002), it is critical to obtain as much information as possible about the anti-angiogenic and anti-tumour capabilities of these compounds. In conclusion, we have demonstrated potent anti-angiogenic properties within both currently identified classes of thalidomide analogue. This is independent of previously described immunomodulatory properties known, including TNF-α inhibition. This information adds to our knowledge concerning these novel and promising therapeutic agents and may help to target compounds to their appropriate clinical setting.
